# Resolution and Argument

**Published:** 1852-01

**Authors:** Elisha Townsend


### ARTICLE II.

### *Resolution and Argument offered at the Last Meeting of the American Society of Dental Surgeons.* By ELISHA TOWNSEND, D. D. S.

*Resolved,* That the objects avowed by this society, at the time of its organization, and the purposes of its founders, were all right, necessary and well-timed, and the measures adopted, honorable, expedient and successful; that, eleven years' experience, fully justifies the hopes entertained of its agency, in advancing the interests of the science, and elevating the general reputation of the profession; and, that its present condition and duties, and its prospects of usefulness, commensurate with its capacities, alike, require an enlargement of its sphere of action, an increase of energy in its efforts, and of freedom and liberality in its spirit.

MR. PRESIDENT:

This society is now eleven years old. It is the oldest of the national associations of this country, formed for the advancement of the various branches of the healing art. It claims the high honor of being the pioneer, in this tract of professional enterprize. Its founders felt so sharply the necessity, and saw so clearly the advantage of professional community, that they ventured upon the untried principle, in a confidence, equally creditable to their intelligence and their zeal. All honor and thanks to them, that we witness, to-day, the happy fulfilment of their generous hopes. Their experiment is our experience, for, the PURPOSES of our organization, so far as time can fulfil them, are already eminently answered. Dr. Horace H. Hayden, the father of our society, and its first President; a man, sir, who was gifted with that prophet-like constitution of mind, which enables its possessor to *foresee* the *results* of great enterprizes, as clearly as their humbler agents can see them, when they arrive; and was, moreover, endowed with that energy in action, which is as essential as insight, to the character of hero and reformer.

Dr. Hayden, sir, when the handful of leading spirits, were assembled at New York, to adopt our constitution, said, that he had resolved, the time should come when the profession and *name* of dentist should be honored, and in the address, which he delivered before us, here, in Philadelphia, at our second annual meeting, ten years ago, in answer to the question, "what rank shall we assume?" replies, in the confidence of inherent right and actual desert, "we assume the title, and claim the privileges, (hitherto denied us,) of being the studious, diligent and successful cultivators of at least one branch of the science of medicine." And, looking forward to the happy working of all the agencies, then enlisted, and among which, he held this society a most important one, he promised our profession, an "elevation that shall command the respect, not only of its own members, but of the medical faculty in every part of the world." I need not refer, now, to the evidences; for the fulfilment of this scripture, is seen and known of all men. The name of dentist *is* honored, the respect of the faculty *is* secured, and our profession is fully recognized, as a legitimate and honorable branch of the great medical family.

It is the agency and capability of this society, which the resolution presents for remark, and I turn from the happy and proud contemplation of our position, in the world of science, to a brief consideration of our societary relations, and the duties growing out of them. In reference to the agency of our association, it seems to me, sir, that its designed efficiency in the improvement, and advanced reputation of our profession, cannot well be questioned.

It is not necessary to the establishment of such claims, that it should be credited with the exclusive, or even a principal share, among the causes of the general prosperity, for which it has been laboring. It is enough to say, that intrinsically, it is capable of a noble service in the cause of scientific progress and professional education, and that it has been, during these years, faithfully and earnestly employed, upon its mission; that it has been busy with the interests, and alive with the sympathies of the great work, and has kept itself abreast of every forward step of the great advancement made, and still stands ready, and fully capable, of all those next steps in progress, which lie before us.

There is great pleasure in the retrospect of such an enterprize as ours. Its mistakes and imperfections are as nothing, in the average of good which it reports for the past, and promises for the future ; nay, such mistakes and imperfections, are the instances which prove the rule, for they show the strength of the principle which could accomplish its aims against all impediments in its subject, and their hindrances besides. A society which had our work to do, and can report such progress as we this day witness, has justified its existence, and deserves the place it occupies, and must, also, be as capable of its future promises, as of those which it has already fulfilled.

Well do I know, that the infancy of all things human, as well as of the human animal, is liable to disease and disturbance, and we can afford to confess the common infirmities for ourselves, for we have lived through them, and have escaped with a sound constitution, and have all the advantages of large and various experience for our future direction.

The early years of such a movement, are entitled to the benefit of this justification or apology, as either may be needed, to cover the complaints of enemies, and the regrets of friends. Childhood is usually a hard struggle for the maintenance of the individual life, and that of our society was one of the hardest. The preamble of our constitution gives conspicuous place among the motives of the organization, to the necessity then felt, of giving "character and respectability to the profession, by establishing a line of distinction between the truly meritorious and skillful, and such as riot in the ill gotten fruit of unblushing impudence and empiricism."

Dr. Hayden, in his characteristic way, compared the mischiefs then to be combated, to "a new race of Canaanites, which must be expelled from our borders." In such circumstances, the chief and highest functions of a young society, like those of human infancy, lie somewhat in advance, awaiting self-adjustment, and more mature conditions for their perfect performance. Yet, even an absorbing care of itself, is in some sort, a necessary fidelity to its prospective purposes. Accordingly, the effort to vindicate and preserve our character and caste, was a leading impulse, and exacted the utmost vigilance of our early membership.

Just then, there was nothing more important, and nothing so immediately pressing; and the error of excess in that direction might be expected, and will, in all candid judgments, be excused. Qualifications for membership usually figure largely in new constitutions; and exclusiveness in the spirit of their management, when it has a warrantable jealousy for the common honor to justify it, is apt, besides, to find many occasions and strong impulses, in the pride and prejudices of opinion, common to all men of strong and decided quality.

Indeed, it is not only a pardonable, but almost a graceful fault, this overweening pride of position, and care for character honorably earned, to be usefully exerted; but it is, also, so natural and plausible, that there is no danger of its *undue* slackness, and there is the less necessity for special care in its maintenance. The time comes to such a society, as to the child with which we compare it, when it may be trusted without extraordinary securities, and may encounter nearer contact with the masses, under the protection of its real strength, and the acquired reputation of it. Besides, it must be recollected, its ultimate business is with the masses, and it must fulfil its vocation, at all events, for, as Dr. Johnson said to the idler, who begged a shilling from him, on the grounds that he *must* live, "that necessity is not so clear as you seem to think, for one that does nothing he was intended for."

The resolution now before us, refers with just pride, to the influence of our society upon the general interests of our profession, but, I do not propose the argument of this proposition, as if it were in controversy, nor do I urge it for the mere purpose of asserting its claims upon the regard of the public, professional and non-professional. I acknowledge, that I feel my full share of pleasure, in the honorable attitude which it occupies in the scientific world, and a happy confidence in its well proved powers of usefulness, but it is for the sake of those powers, and the hope that there is in them, that I would assure myself of its worthiness and rejoice in it.

My impression is, sir, that beside and after all that individuals can do in discovery and improvement, and in the propagation of such improvement through the means of private pupilage—after all that our excellent Dental College can do, in giving the highest educational advantages to their public classes, and after, and besides, all that our invaluable dental periodicals can achieve in the collection and circulation of professional knowledge, and in exciting its development: there still remains important and appropriate work for organizations such as ours, whether they be national or state, or limited to lesser districts; and, that their importance is also in proportion to the extent of territory, and variety and number of members which they embrace. All the formal methods of education mentioned, have their special adaptation to use, and none of them can supply the place of another, nor take rank of it in importance. Each in its own way, is alike necessary to the general end; but they all lack what a national organization of practitioners is capable of supplying; they want that combination and unity, and that attitude of supervision, in virtue of a general representation, which serves to ascertain the idea, and direct and sustain the drift of professional advancement.

The private instructor imparts to his pupils, along with the elements that are common, all that is peculiar and individual in his own notions and methods, but without the criticism and connection of a broad comparison with others. The college teaches through its men, selected for their eminence and ability, all whatsoever can be so taught and received; but the period of technical pupilage and primary education stops, aye, stops at the very threshhold of the profession. Our periodical papers are indeed invaluable masters in the work of that mutual or second education, which adepts can interchange with their equals; but with all the reciprocity of instruction, and facility of communication which they possess, and which gives them their great value, they yet lack the peculiar power of the associative principle, and that superiority of influence which the personal communion of *men*, has over the mere communion of mind through intermediate channels. Authorship and direct intercourse touch their subjects very differently ; writers, while they teach readers, derive for themselves from their responsibilities, all the training and influence of scrutiny and trial at the bar of opinion, but the readers, who need such influence most, escape it, and in escaping, lose all the benefits of emulation ; for between them and their book makers there is no sort of competition, and no mutuality. But personal contact in associative movements brings the men of both classes together, and excites them equally with all the force of a full responsibility of all parties, to the same expectations and duties.

It provides for such equality, and provides for such reciprocity of relations, as must act upon the younger and inferior in the happiest manner, while, in fact, it gives the tone and pitch of the highest and best to the whole fraternity. Men balance better when they meet in person, than when they stand edgewise to the world to show only their strongest points ; and the challenge to equality of average usefulness, comes, graced and commended among professional men, (otherwise differently distinguished,) by those amenities of personal intercourse in which, at least, every respectable man has as much to give as to receive from his neighbor, and by all those other differences between men that make the most common necessary to the completeness of the most distinguished. You, Mr. President, for instance, have a potent voice ; but I have a testing vote—you have an advance idea, I have a correcting experience—you have shining talents for speculation, I balance you with eminent opportunities for observation ; and so the man that makes a figure exchanges dependency with the man most conversant with facts, and the great balance holds the truth and the excellence of all, for the use of all. Moreover, there is in the feeling of this fellowship in the highest things, the turn of another coil of the great chain of sympathy, which holds men to the highest apprehension of their professional duty, and to the faithfulest performance of it. Again, an association of the mature men of a profession, has another use, that works wider than its own limits, which is well worthy of our regard. It affords the non-professional public a pledge of guardianship over the general interests, and secures for the profession the confidence that the known watchfulness of all for the benefit of each, is calculated to inspire. Every one feels sure that men, so related to each other, must not only contribute of their best for their credits' sake to the common stock, but must, in the spirit of such emulation as they have, whether generous or selfish, study and strive to make it as worthy as possible of acceptance and display. In every way, indeed, the blessing of human brotherhood, and the force of universal helpfulness, are in it. Empiricism of every grade and form is expelled by the liberal spirit of fraternity ; and selfishness and individualism, caught in their own craft, gladly throw open their doors to exchange their farthing rush-lights for the broad sunshine of the open day ; and over all, like a general providence, or a controlling destiny, opinion, which it moulds, leads all effort and aspiration by settling all pretension in science by the highest ideas which get general allowance and authority before the public. Such are some of the capabilities of association—very imperfectly arrayed—very feebly expressed, but imposing enough in any presentment to justify all we claim for it.

And now it remains for us to make it yield the highest results of which it is capable, and to effect this, we must make the policy and spirit of the movement correspond to the purpose, with such *liberal* accommodations to the condition of things around us, as the great principle of our enterprise will allow. I speak as I have heretofore spoken—encouraged by the kindness of construction, and the ready sympathy and support accorded to me by my brethren. I speak for the enlargement of our borders, the extension of our influence, by every means that can favor such ends, and with as little restraint and scruple as self-respect will, in any wise admit. The profession boasts a large number of worthy practitioners, who have never been in membership with us, though we are on the best terms of personal respect and professional intercourse with them: now there is nothing in our constitution, or in the terms of communion, which should bar out a single legitimate practitioner of dental science in the United States. We are not a close corporation of conservatives, but in spirit and in fact, a company of men zealous for progression, and free in our relations and movements, as the love of truth and progress can make us. We are conscious of no intended impediment to the widest, broadest, freest participation with us by all the regular brotherhood of the profession. But, if we are misapprehended in our design, or held delinquent in any duty or courtesy, which might make our association more attractive, it is right for us to remove all misapprehension by the most explicit manifestation of our cordiality to the fraternity, and by earnest action in accordance with it. The resolution submitted, seems to me appropriate for the purpose of such declaration, and the general demand of the membership will most unquestionably illustrate and sustain it in practice. Closely connected with our sentiment and bearing, as a society, towards the dentists of the country, not in connection with us, is that of the conduct of the older and more distinguished among us, in their general professional relations with the younger and less eminent practitioners of our art. This topic does not directly rise out of the resolution, though it has some relevancy, and it may even be pressing quite to the verge of my license, to touch it, however respectfully in spirit and manner.

But, I have always found the candor and forbearance of the men whom I am now addressing, quite worthy of the earnestness with which they were approached, and I am emboldened thereby to put my brethren to the question upon this point. Have we as cordially as we might, kept open, wide open, the approaches that secure confidence and fraternity between all the grades and varieties of professional men? The best methods of education on dental science and art, have not been long open to any, and are not yet conveniently accessible to all. Moreover, our profession, in all its branches, is not to be fully learned by the ordinary opportunities which the best private practice can afford to the pupil, and from other causes besides, young men capable of the best things, as well as others less worthy, must needs enter upon business, in a condition of comparative incompetency: now, it is required of us all, every one of us, to answer, whether we have been considerate and kind enough in the premises; whether we have magnanimously laid aside as much of the difference which better opportunities and greater experience has fortunately afforded to us, as would reconcile them to us, and fit us for our duty to them, and so meet them in the liberal spirit that real superiority always wears, when it is also above selfishness, pretension and fear. I have spoken of association on the larger scale which the American Society presents, and I would now gladly press the value and duty of private professional association, to the full extent that freedom of communication between neighboring practitioners would require, and for the purposes of the frankest intercourse and interchange of observation and mutual instruction. The difficulty and delicacy of such freedom are not so great in our department, as in that of medicine, and some of the operations of general surgery, for our patients are not in sick rooms, but we can make appointments to meet our own convenience, and postpone treatment sufficiently to bring good cases into use for the common benefit: we can select our cases, and appoint our times almost at will—and there is nothing to hinder periodical reunions of men in full practice, with their young friends, for opportunities which could be made available and invaluable to all parties, and most so to him that imparts the most. In a word, let us liberalize our professional relations—enlarge and enrich its brotherhood, and give the *heart* of the man its natural place and power through the whole range of the relations and duties of our professional vocation. And now, sir, while the ideas and aspirations appropriate to these views of the cultivation of our science, are all aglow with the fraternal feelings which they so thoroughly awaken, permit me, as one of the committee of arrangement for the meeting of the convention, to bid the American Society welcome to our city, and to assure them of an earnest and cordial hospitality, in return for the high gratification which their presence affords to their friends, professional and personal, in the city of brotherly love.

